# LC–TOF-MS/MS and GC-MS based phytochemical profiling and evaluation of wound healing activity of *Oroxylum Indicum (L.)* Kurz (Beka)

**DOI:** 10.3389/fphar.2022.1050453

**Published:** 2022-11-22

**Authors:** Ferid Abdulhafiz, Mohd Farhan Hanif Reduan, Anwar Hazim Hisam, Ibtihal Mohammad, Ikarastika Rahayu Abdul Wahab, Fathin Faahimaah Abdul Hamid, Arifullah Mohammed, Muhammad Luqman Nordin, Rumaizi Shaari, Luqman Abu Bakar, Zulhisyam Abdul Kari, Lee Seong Wei, Khang Wen Goh, Muhammad Rajaei Ahmad Mohd Zain

**Affiliations:** ^1^ Faculty of Agro-Based Industry, Universiti Malaysia Kelantan, Kota Bharu, Malaysia; ^2^ Faculty of Veterinary Medicine, Universiti Malaysia Kelantan, Kota Bharu, Malaysia; ^3^ Faculty of Data Science and Information Technology, INTI International University, Nilai, Malaysia; ^4^ Department of Orthopaedics, School of Medical Sciences, Universiti Sains Malaysia, Kubang Kerian, Malaysia

**Keywords:** natural products, phytochemicals, beka plant, metabolic profiling, wound healing, herbal medicine, inflammation

## Abstract

**Background:** Beka (*Oroxylum indicum* (L.) Kurz) has been used as a culinary herb and natural remedy by the local communities in Malaysia. The leaf of *O. indicum* is traditionally used for the treatment of diarrhea, high blood pressure, and improving digestive health.

**Objectives:** The present study was conducted to evaluate the phytochemical constituents and wound healing properties (*in vitro* and *in vivo* models) of aqueous and ethanol extracts of *O. indicum* leaves.

**Methods:** The total phenolic (TPC) and total flavonoid (TFC) contents in the plant extracts were determined by the spectrophotometric methods. Further, the extract was characterized by Liquid Chromatography Time-of-Flight Mass Spectrometry (LC-TOF-MS/MS) and Gas Chromatography-Mass Spectrometry (GC-MS). The wound healing activity was assessed using the *in vitro* scratch wound-healing assay and *in vivo* excisional wound model.

**Results:** The results show the ethanol leaves extract had the higher TPC (164 mg GAE/g) when compared with the aqueous leaves extract (30 mg gallic acid equivalents/g). The ethanol leaves extract was also found to have higher TFC (101 mg Catechin equivalents/g) than the aqueous leaves extract (76 mg Catechin equivalents/g). The ethanol leaves extract was then used for further chemical analysis. The LC–TOF-MS/MS analysis showed that the leaves extracts of *O. indicum* contains many important compounds such as Orientin, Chrysin, Pinoquercetin, Cupressuflavone, Puerarin xyloside, Forsythiaside and Paederoside. In GC-MS analysis, 19 compounds were identified in ethanolic leaves extract. The wound healing studies shows that *O. indicum* has promising wound healing activity by increasing the rate of wound contraction significantly (*p* < 0.05).

**Conclusion:** In conclusion, the present study showed that *O. indicum* leaf contains important phytochemicals and the wound healing potential of the *O. indicum* extract may probably be as a result of the presence of various phytoconstituents.

## Introduction

The wound healing process is described as a complex biological process where multiple and interrelated pathways are activated to induce wound repair (Asadi et al., 2013). The wound healing process starts immediately after the injury, generally wound healing process is characterized into three phases: 1) inflammatory phase, consisting of homeostasis and inflammation; 2) proliferative phase, consisting of granulation tissue formation, epithelialisation and contraction; and 3) the remodelling phase (Morales-González et al., 2022). For the wound healing process to occur, it is important to undergo a process that consists of a sequence of molecular and cellular events for the tissue damage to be fixed and restore. These events occur through the integration of dynamic processes involving soluble mediators, blood cells and parenchymal cells (Tottoli et al., 2020). It also involves partially overlapping phases, as some of the factors such as contaminated wound cause prolonged of certain phases, usually the inflammatory phase, which lead to delays in the wound healing process (Tottoli et al., 2020). For wound management or method in treating wounds are debridement, irrigation, antibiotics, tissue grafts and proteolytic enzymes, but these methods have disadvantages and undesirable side effects.

Herbs have been used for both culinary and medicinal purposes for many years (Khanal et al., 2021). Consumption of herbs may help to treat or prevent several diseases such as cancer, diabetes, high cholesterol levels, cardiovascular, and inflammatory diseases (Khanal et al., 2021; Sachdeva et al., 2022). Besides serving as a culinary and medicinal purpose, herbs have been also a valuable source for the production of novel pharmaceutical drugs, food preservative agents and cosmetics products (Gurib-Fakim, 2014; Bailly, 2021; Abdulhafiz, et al., 2022). Recently, the demand for herbal medicine and natural products has increased massively. The increased patronage and interest on herbal medicine has been attributed to several reasons such as safety claims, growing preference for natural therapies, high cost and adverse side effects of modern drugs (Ekor, 2014; Abdulhafiz et al., 2020b; Alsamri et al., 2021; Maher et al., 2021a).

Plants contains various chemicals, also known as phytochemicals, and are responsible for beneficial or biological effects. These chemicals are stored in their various organs such as fruits, seeds, leaves, stems, roots, bark etc. Phytochemical contents in any plant (even within the species) vary from place to place as it is affected by various factors such as environment, rainfall, temperature and growing conditions. Recently, more attention has been focused on identification and quantification techniques to get useful chemical information (fingerprint) in crude extracts from medicinal herbs. This approach has helped to a better understanding of herbs and herbal products (Goodarzi et al., 2013; Chen et al., 2016; Abdulhafiz et al., 2020a; Reduan et al., 2020). Several analytical methods have been developed and used by various researchers around the world (Tistaert et al., 2011; Saini et al., 2016; Rahman et al., 2018; Kharbach et al., 2020; Maqsood et al., 2020; Maher et al., 2021b). One of the most recent technique for generating herbal fingerprints is LC-TOF-MS/MS. This analytical tool has high separation capacity, sensitivity, detects a wide range of chemicals in a short time by its TOF analyzer and provides accurate mass measurement for metabolite identification (Alaerts et al., 2010; Wu et al., 2022) and this method allows the biomarker discovery where the candidate markers are likely to present in smaller quantity (Metz et al., 2007; Lei et al., 2011; Gowda and Djukovic, 2014; Rainville et al., 2014; Want, 2018).

*Oroxylum indicum* (L.) Kurz belongs to the family Bignoniaceae and is widely distributed throughout Asian counties, including Malaysia, Thailand, Indonesia, India, Vietnam, Philippines, China, Taiwan, and Japan. It is an important culinary herb with several medicinal and culinary purposes (Swargiary and Daimari, 2020). The parts used in traditional medicinal practices are the fruit, seed, leaves, stems, bark and roots. Although, all parts of the plant are being used in traditional medicine, some of them still remain unexplored. Some pharmacological studies reported the medicinal values and phytochemical content of *Oroxylum indicum*, some of which include antidiabetic (Singh and Kakkar, 2013; Sun et al., 2018), antiadipogenic activity (Hengpratom et al., 2020) and anticancer (Yoganarasimhan, 1996). Therefore, this study was conducted to evaluate the phytochemical constituents and wound healing properties (*in vitro* and *in vivo* models) of aqueous and ethanol extracts of *O. indicum*.

## Methods

### Chemicals and reagents

Chemicals and solvents such as sodium nitrate, aluminum chloride, sodium carbonate and ethanol (95%) were purchased from local vendor. Folin-Ciocalteu was purchased from Sigma-Aldrich (United States). Distilled water, whatman No.1 filter paper, and aluminum foil were obtained from the laboratory.

### Plant sample collection

The fresh leaves of *Oroxylum indicum* (approximately one month old) were obtained from the local market in Kota Bharu, Kelantan, Malaysia. The plant part was identified by a botanist from Herbarium, Universiti Kebangsaan Malaysia, Selangor, Malaysia. A voucher specimen (ID025/2020) was deposited at the herbarium for reference. The leaves were given cut from leaf axil and separated from the main stem carefully. The leaves sample were then washed with tap water, dried in the open air, and then ground into powder using an electrical blender (Milux MCB-905, Milux Sdn Bhd, Selangor, Malaysia). The powder was stored in air tight plastic container until further use.

### Preparation of sample extract

Sample extraction was carried out as described by Abubakar and Haque (2020) with some modifications. Briefly, powdered plant samples about 150 g were macerated for 72 h with 1.5 L of distilled water or 95% ethanol solvent at the room temperature. After 72 h of soaking, the extracts were filtered through Whatman No.1 filter paper. The filtrates were then concentrated to dryness using a rotary evaporator at 40°C. The crude extracts were then stored at 4°C until further use.

## Preliminary phytochemical study

### Determination of total phenolic content

TPC was determined by using Folin-Ciocalteu reagent spectrophotometrically following the procedure of Abdulhafiz et al. (2020b) with minor modification. Briefly, 100 µL of aqueous or ethanolic extract (500 μg/ml) was mixed with 1 ml of FC reagent (10%). After 10 min, 1 ml of sodium carbonate (7.5%, w/v) was added, mixed and incubated in the dark for 90 min. The absorbance was measured using a spectrophotometer (UV-1900, UV-Vis Spectrophotometer, Shimadzu Co. Japan) at λ_max_ = 750 nm against the blank solution containing 100 µL of 95% ethanol, 1 ml of FC reagent (10%) and 1 ml of sodium carbonate (7.5%). The samples were prepared in triplicate for each analysis and the mean value of absorbance was obtained. TPC was calculated from a calibrated curve, using gallic acid as standard (12.5–100 μg/ml). The concentration of TPC was expressed as mg of gallic acid equivalents per gram of dry weight (mg GA/g extract).

### Determination of total flavonoid content

TFC in the plant extracts was determined by the aluminum chloride colorimetric method following the procedure of Fattahi et al. (2014) with a minor modification. One hundred microliters of aqueous or ethanol extract (1 mg/ml) or standard were added to 4 ml of sterile distilled water. Then 0.3 ml of 5% sodium nitrate solution (w/v) was added. After 5 minutes, 0.3 ml of 10% aluminum chloride solution (w/v) was added. In 6 min, 2 ml of 4% (w/v) NaOH was added to the mixture. Then, the mixture was diluted with 1 ml distilled water and mixed thoroughly. The absorbance was measured at λ_max_ = 518 nm immediately. Standard Catechin was used to construct the calibration curve (50–250 μg/ml). The concentration of TFC was expressed as mg of Catechin equivalents per g of dry extract (mg CE/g d.wt extract).

### Gas chromatography-mass spectrometry (GC-MS) analysis

The GC-MS analysis of the ethanolic leaves extracts of Beka was carried out by Gas Chromatography-Mass Spectrometry, GC-MS (Perkin-Elmer /Clarus 600T). The Perkin Elmer Clarus 600 GCMS was coupled to TurboMatrix Headspace Sampler 40, run on GC-MS column Elite 5MS (30 m × 250 mm x 0.25 µm). The GC-MS condition was set at the Initial oven temperature of 70°C and raised to 76°C at the rate of 1°C/min and was maintained for 1 min. Then, the oven temperature was raised to 300°C, at the rate of 6 °C/min, and was maintained for 5 min. Injection temperature was ensured at 250°C and helium flow rate 1 μL/min. Ionization voltage 70 eV, split mode as 50:1. The mass spectra range was set at 0–800 (m/z). Compound identification was carried out using the National Institute of Standards and Technology (NIST), version 2.0 reference library and literature data.

### Liquid chromatography-time of flight-mass spectrometry analysis

The separation analysis was carried out using Thermo Scientific C18 reversed-phase column of Acclaim^™^ Polar Advantage II, 3 × 150 mm, 3 µm particle size on a Dionex Ultimate 3,000 ultra-high-performance liquid chromatography (UHPLC) system (Thermo Fisher Scientific, Waltham, MA, United States), maintained at 40°C. The injection volume was 3 µL. The mobile phase consisted of 0.1% formic acid in water (solvent A) and High purity acetonitrile (solvent B), at a flow rate of 0.4 ml/min for a total run time of 22 min. The gradient elution applied was: 5% A (0–3 min); 80% B (3–10 min); 80% B (10–15 min) and 5% B (15–22 min). The extract was analyzed by reverse phase LC-MS for their metabolites contents. The mass spectrometry (MS) analysis was carried out using MicrOTOF-Q III (Bruker Daltonik GmbH, Bremen, Germany) in the electron spray (ESI) negative mode; capillary voltage of 4000 V, nebulizer pressure: 2.0 bar; drying gas: 8 L/min at 300°C. The data acquisition was performed using Compass Data Analysis software version 4.3 and the mass range was set at the mass-to-charge ratio 50–1,000 m/z. The metabolites were identified based on accurate mass and MS/MS fragments by searching MetFrag (https://msbi.ipb-halle.de/MetFragBeta/, accessed 18 January 2022) and PubMed (https://pubmed.ncbi.nlm.nih.gov/, accessed on 18 January 2022) an online public-databases. In addition, previously published literatures were utilized in comparing fragment pattern with their findings. The secondary metabolites in the leaves of *O. indicum* were analyzed by untargeted analysis.

## Wound healing properties of the leaves extracts of *oroxylum indicum*

### *In vitro* scratch wound-healing assay

HaCaT cells were acquired from the Virology Laboratory, Faculty of Veterinary Medicine, Universiti Malaysia Kelantan. The cell lines were thawed and well maintained. The cells were cultured and maintained in high glucose Dulbecco’s Modified Eagle Medium (DMEM) premixed with 10% fetal bovine serum and antibiotics (Streptomycin 100 μg/ml and Penicillin 100  U/Ml) in a humidified 5% CO_2_ incubator at 37°C.

The migration rates of HaCaT cells were assessed by the scratch assay method. The cell density of “2 × 105 cells” were spread into each well of a 24-well plate and incubated with complete medium at 37°C and 5% CO_2_. After 24 h of incubation, the monolayer confluent cells were horizontally scratch with sterile P200 pipette tips. Then, the debris was removed by washing with Phosphate-buffered saline (PBS). After that, the cells were treated with ethanolic and aqueous extracts of *O. indicum* leaf with various concentration (12.5 μg/ml, 25 μg/ml, and 50 μg/ml) by diluting with serum-free Dulbecco’s modified Eagle’s medium (DMEM). The cells without treatment and treatment with allantoin (Sigma Aldrich, Germany) (50 μg/ml) was used as the control and positive control, respectively. Then, the scratch induced that represented wound was photographed at 0 h by using phase-contrast microscopy at ×40 magnification at 0 h before incubated with the ethanolic and aqueous extracts of *O. indicum* leaf. After 24 h of incubation, the second set of images were photographed. The images were analyzed and measured using “ImageJ” software to determine the wound closure and compared with the values obtained at 0 h. An increase in the percentage of wound closure indicated the migration of cells. Experiments were performed in a triplicate manner.

$$Migration\;Rate\;\left(\%\right)=\frac{\left(Measurement\;at\;0\;h-Measurement\;at\;24\;h\right)}{Measurement\;at\;0\;h}$$

### Experimental animals

A total of twenty male rats at 8 weeks old age were obtained from Animal Research and Service Centre, Universiti Sains Malaysia (ARASC USM), Health Campus, Kubang Kerian, Kelantan. These animals were randomly selected and caged individually within a polycarbonate cage. All the rats were kept within a standard laboratory condition with a temperature (24 ± 2°C), relative humidity (45 ± 5%) and with a lightning control of 12 h daylight and 12 h dark cycle. Food and water were given *ad libitum*. The bedding used were wood shaving and changed daily. All rats undergo acclimatization for five days prior to the experiment is started.

### *In vivo* excisional wound model

The study was conducted with the approval from Institutional Animal Care and Use Committee (IACUC) of Faculty of Veterinary Medicine, Universiti Malaysia Kelantan (IACUC number: UMK/FPV/ACUE/FYP/14/2021). The wound was inflicted in all rats at the initial of the experiment (day 0). All the rats were anaesthetized using a combination of xylazine hydrochloride and ketamine with the dosage of 5 and 30 mg/kg respectively through intramuscular injection prior to wound excision. Skin preparation was conducted in an aseptic manner. A full thickness of the squared-shape excision wound with 2.5 cm long and width was excised with the use of toothed forceps and pointed scissor (Thakur et al., 2011).

A total of 20 rats were randomly assigned into 5 groups of 4 rats in each group. The extract at different concentrations (1%, 3% and 5% w/w) was applied topically on the excisional wound of rats at group 1, group 2 and group 3. The positive control group which group 4 received Sudocrem cream while no treatment was given to the negative control group (Group 5). Wound size was measured at days 0, 4, 8, and 14 post-wounding. The initial wound size (day 0) was measured at 3 h post-infliction. The wound size (2 mm) was determined using a calliper. Wound contraction, which contributes to wound closure, was expressed as a reduction in the percentage of the original wound size from the initial day of the experiment until the day of complete wound closure. It is used to calculate the degree of wound healing (Thakur et al., 2011). In each day interval, the wound size was considered as a non-healed area, which was subtracted from the initial wound size to obtain healed area. The rate of wound contraction was determined using the formula; Rate of wound contraction (%): Healed area/Initial wound size x 100%.

### Statistical analysis

The data were analyzed by one-way ANOVA to identify significant differences among the treatment/control groups (*p* < 0.05) using SPSS v. 21.0 statistical software program. All data were presented as mean ± standard deviation.

## Results

### Total phenolic and flavonoid content in *O. indicum* extracts

**FIGURE 1:**
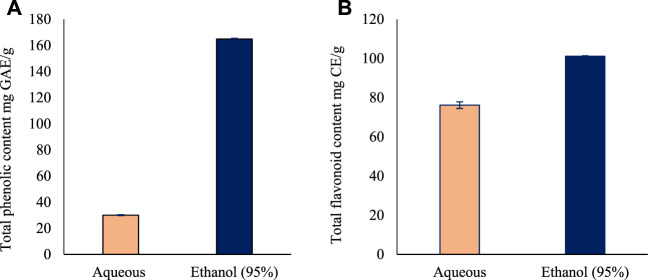
The total phenolic and flavonoid content of the leaves extracts of *Oroxylum indicum*. **(A)** Total phenolic content in *Oroxylum indicum* leaf aqueous and ethanolic extracts. **(B)** Total flavonoid content in *Oroxylum indicum* leaf aqueous and ethanolic extracts. Results are mean ± SD (*n* = 3).

The total phenolic content for aqueous and ethanol leaves extracts of *O. indicum* were estimated by Folin Ciocalteu’s method using gallic acid as standard. A linear calibration curve of standard gallic acid with r^2^ value of 0.999 was obtained. The total phenolic content of the leaves extracts were measured using GAE equation of *y = 0.0035x - 0.0369*. Among the two solvents extract, the ethanol (95%) leaf extract had the higher TPC (164.9 ± 0.1 mg GAE/g) compared with the aqueous leaves extract (30.1 ± 0.1 mg GAE/g) (Figure 1A).

The total flavonoid content for aqueous and ethanol leaves extracts of *O. indicum* were estimated by aluminum chloride colorimetric method using catechin as standard flavonoid compound. A linear calibration curve of standard gallic acid with *r* ^*2*^ value of 0.9825 was obtained. The total flavonoid content of the leaves extracts were measured using GAE equation of *y = 0.0006x—0.0197*. Among the two solvents extract, the ethanol (95%) leaves extract had the higher TFC (101.2 ± 1.6 mg Catechin equivalents/g) followed by the aqueous leaf extract (76.2 ± 0.2 mg Catechin equivalents/g) (Figure 1B).

### GC-MS analysis of the ethanolic leaf extract of *Oroxylum indicum*

**FIGURE 2:**
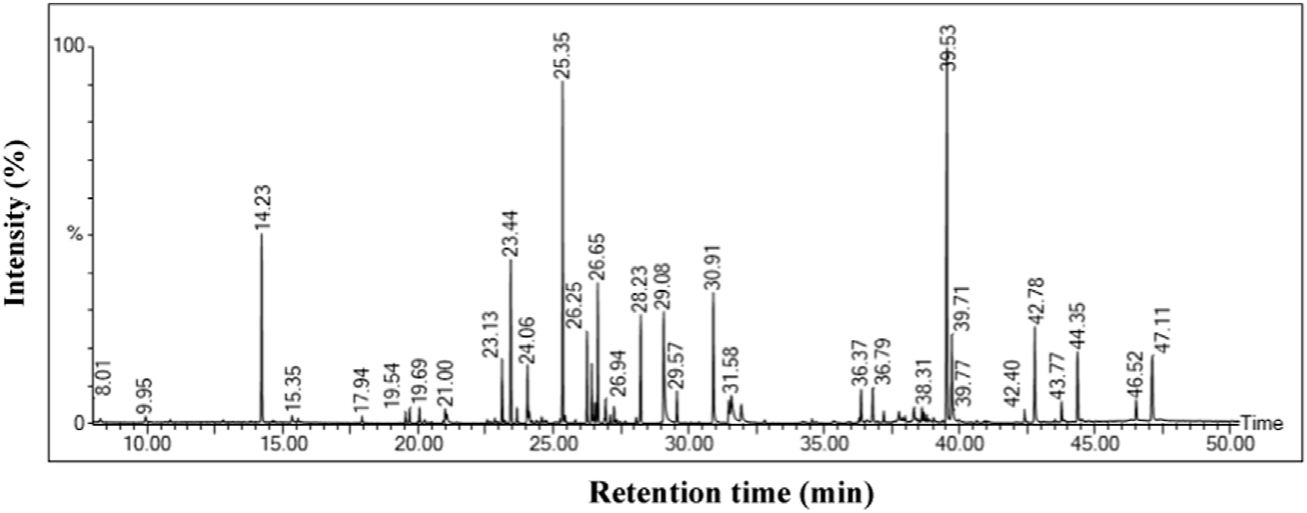
GC-MS chromatogram of *Oroxylum indicum* leaf extract.

Untargeted GC-MS analysis was performed to analyze and tentatively annotate the extracted metabolites. The GC-MS analysis of an ethanolic extract of the *O. indicum* showed the presence of several compounds. The GC-MS chromatograms of the ethanolic extract are shown in Figure 2. The retention time, name of the phytochemicals annotation, and other GC-MS profiles are presented in Table 1.

**TABLE 1:** GC-MS analysis revealed the presence of phytochemical components in ethanol leaf extract of *Oroxylum indicum*.

No	Retention time (min)	Compound name	Area (%)	Molecular Weight	Molecular formula
1	9.948	Pentanoic acid, 2,2,4-trimethyl-3-carboxyisopropyl, isobutyl ester	0.307	286.41	C_16_H_30_O_4_
2	10.862	Trisiloxane, 1,1,1,5,5,5-hexamethyl-3,3-bis[(trimethylsilyl)oxy]-	0.093	408.9	C_9_H_27_O_3_Si_4_
3	14.235	Trimethylsilyl ether of glycerol	5.839	308.64	C_12_H_32_O_3_Si_3_
4	15.350	Butanedioic acid, bis(trimethylsilyl) ester	0.327	262.45	C_10_H_22_O_4_Si_2_
5	19.545	3,8-Dioxa-2,9-disiladecane, 2,2,9,9-tetramethyl-5, 6-bis[(trimethylsilyl)oxy]-, (R*,S*)-	0.254	234.48	C_10_H_26_O_2_Si_2_
6	23.666	l-(-)-Arabitol, pentakis(trimethylsilyl) ether	0.442	513.05	C_20_H_52_O_5_Si_5_
7	25.450	Benzoic acid, 3,4-bis[(trimethylsilyl)oxy]-,trimethylsilyl ester	0.165	370.66	C_16_H_30_O_4_Si_3_
8	26.253	D-Fructose, 1,3,4,5,6-pentakis-O-(trimethylsilyl)-,O-methyloxime	2.562	570.1	C_22_H_55_NO_6_Si_5_
10	26.547	d-Galactose, 2,3,4,5,6-pentakis-O-(trimethylsilyl)-,o-methyloxyme, (1E)-	0.649	541.1	C_21_H_52_O_6_Si_5_
11	26.888	d-Galactose, 2,3,4,5,6-pentakis-O-(trimethylsilyl)-,o-methyloxyme, (1Z)-	0.117	569.2	C_22_H_55_NO_6_Si_5_
12	28.235	Galacturonic acid, pentakis(trimethylsilyl)-	3.103	555.0	C_21_H_50_O_7_Si_5_
13	29.082	Hexadecanoic acid, trimethylsilyl ester	5.676	328.6	C_19_H_40_O_2_Si
14	30.914	Phytol, trimethylsilyl ether	4.724	368.7	C_23_H_48_OSi
15	31.486	9,12-Octadecadienoic acid (Z,Z)-, trimethylsilyl ester	0.913	352.6	C_21_H_40_O_2_Si
16	31.582	à-Linolenic acid, trimethylsilyl ester	2.424	350.61	C_21_H_38_O_2_Si
17	31.949	Octadecanoic acid, trimethylsilyl ester	0.941	356.7	C_21_H_44_O_2_Si
18	38.319	Chrysin, bis(trimethylsilyl) ether	0.701	398.6	C_21_H_26_O_4_Si_2_
19	39.534	Hexadecanoic acid, 14-methyl	12.60	372.7	C_18_H_36_O_2_

### LC-MS/MS metabolic profile of the ethanolic leaf extract of *Oroxylum indicum*

The LC-TOF-MS/MS analysis results from *O. indicum* crude extract are shown in Table 2. Annotation was carried out based on the outcome of LC-TOF-MS/MS, the information from the online databases and fragmentation in the previous literature. A total of 12 compounds were detected and summarized along with their retention time, molecular ion and fragmentation data. The MS and MS/MS peak chromatograms and chemical structures present in the *O. indicum* are shown in Table 2.

**TABLE 2:** LC-TOF-MS/MS data-negative ion mode for tentative compounds identification in *Oroxylum indicum* leaf extract.

Peak no.	Retention time (min)	Formula	MS ion (m/z) [M-H]-	MS/MS (m/z) fragment ions	Mass Error	Tentative identification	Chemical classification
P1	8.5	C_32_H_22_O_10_	565.1583	209, 239, 269, 274, 286, 299, 306, 311	0.24	Ginkgetin	Biflavones
P2	8.7	C_26_H_28_O_13_	547.1479	337, 367, 415, 457, 465	0.25	Puerarin xyloside	Flavonoids
P3	8.8	C_29_H_36_O_15_	623.1970	161, 162, 415, 422, 461, 468, 469, 476	0.22	Forsythiaside	Flavonoids
P4	9.1	C_21_H_22_O_10_	433.1162	125, 167, 168, 173, 197, 201, 206	0.30	Phlorizin chalcone	Flavonoids
P5	9.2	C_21_H_20_O_11_	447.0959	284, 285, 286, 289, 440	0.29	Orientin	Flavonoids
P6	9.8	C_9_H_16_O_4_	187.0974	116, 123, 126, 128, 129, 143, 146	0.60	Azelaic acid	Dicarboxylic acids
P7	10.0	C_21_H_18_O_12_	461.0765	253, 283, 285, 286, 288, 289, 290	0.28	Luteolin 7-O-glucuronide	Flavone glycoside
P8	10.6	C_18_H_22_O_11_S	445.0810	267, 269, 270, 274, 384, 391	0.30	Paederoside	Glycoside
P9	11.3	C_16_H_12_O_7_	315.0526	136, 141, 144, 145, 300, 301, 306, 312	0.39	Pinoquercetin	Flavonoids
P10	11.5	C_15_H_12_O_5_	271.0633	125, 128, 145, 148, 149, 253, 258, 263	0.44	Naringenin chalcone	Flavonoids
P11	12.5	C_30_H_18_O_10_	537.0878	220, 225, 243, 244, 245, 283, 391	0.26	Cupressuflavone	Flavonoids
P12	13.2	C_15_H_10_O_4_	253.0532	143, 165, 181, 185, 209, 211	0.47	Chrysin	Flavonoids

According to Table 2, flavonoid account for a large proportion of the components detected in the extract of *O. indicum*. In this way, Peaks 1–5, 9, 10, 11, and 12 were tentatively identified as flavonoid compounds based on their MS/MS spectra, as shown in Table 2.

Peak 1 presented the [M-H]-ion *m/z* 565, and its MS/MS fragment ions at *m/z* 209, 239, 269, 274, 286, 299, 306, and 311 (Table 2). It was identified as Ginkgetin by comparing with those ions recorded in Metfarg. As shown in Table 2, Peak 2 assigned as Puerarin xyloside by comparing with literature reported previously and presented [M-H]—ion at *m/z* 547 and its typical MS/MS fragment ions at *m/z* 337, 367, 415, 457, and 465 by the continuous loss of water molecules (Lin et al., 2020). Furthermore, the fragmentation pathways were reported by Zhang and colleagues (Zhang et al., 2005). Peak 3 displayed the parent ion at *m/z* 623 [M-H]-and its MS/MS fragment ions at *m/z* 161, 162, 415, 422, 461, 468, 469, 469, and 476. The fragmentation pattern (typical product ions at *m/z* 461 and *m/z* 161) confirmed its annotated as Forsythiaside compound (Ye et al., 2010; Varga et al., 2019). Its chemical structure and fragment patterns is presented in Table 2. Peak 4 showed the parent [M-H] - ion *m/*z 433, and produced daughter ions at *m/*z 125, 167, 168, 173, 197, 201 and 206. The parent ion at *m/*z 433 [M-H]^–^ was characterized as Phlorizin providing fragment patterns such as *m/*z 206 (aglycone), 201, 197, 173, 167 and 125 after a neutral loss of (227 Da) (Balázs et al., 2012). Phlorizin has several beneficial effects on human health. Peak 5 showed the [M-H]^–^ ion at *m/z* 447 and the fragment ions at *m/z* 284, 285, 286, 289, and 440. The major fragment of *m/z* 286 was consistent with the fragmentation pattern of orientin (Balázs et al., 2012; Otify et al., 2015; Sulaiman and Balachandran, 2016).

For peak 6, the parent ion at m*/z* 187 was annotated as Azelaic acid as it presented major fragments with *m/z* 116, 123, 143, and 146. This finding agreed with previous reports (Schallreuter and Wood, 1990; Chen et al., 2015; Fialovaa et al., 2015; Zengin et al., 2018). The [M-H]^–^ ion at *m/z* 461 and its fragments at *m/z* 253, 283, 285, 286, 288, 289, and 290 has been assigned as Luteolin 7-O-glucuronide (flavone glycoside compound) according to the reference literatures (Fialovaa et al., 2015; Ibrahim et al., 2015; Sulaiman and Balachandran, 2017). The peak with *m/z* 445 (Peak 8) presented three major fragments with *m/z* 274, 384, and 391 and it was tentatively assigned as Paederoside. The Peak 9 with m/z 315 showed product ions at *m/z* 136, 141, 306, and 312. The fragments suggest that the molecule is Pinoquercetin on the basis of online database information. Peak at m/z [M-H]– 271 (Peak 10) was proposed to be Naringenin chalcone as it displayed major fragments with *m/z* 125, 149, 253, and 258, 263. Peak 11 was tentatively deduced for the *m/z* 537 and its daughter fragments at *m/z* 220, 283, and 391 according to the reference literature (Shan et al., 2018; Zhuang et al., 2018). Peak 12 (*m/z* 253) with its product ions of *m/z* 143, 165, 185, and 211, was annotated as chrysin according to the reference literature (Chen et al., 2003; Rai et al., 2022).

### Effect of *O. indicum leaves extracts on scratch wound healing in HaCaT cells*

**FIGURE 3:**
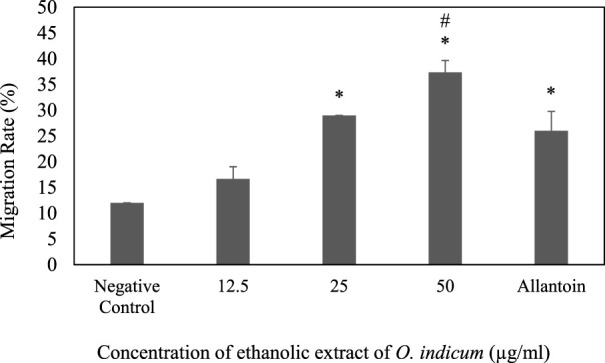
Effect of ethanolic extract of *Oroxylum indicum* on HaCat cell migration. Results are mean ± SD (*n* = 3). The symbol * denotes the significantly (*p* < 0.05) difference between the treated and negative controls. The symbol # denotes the values are significantly (*p* < 0.05) different from the positive control group.

The effect of ethanolic leaves extracts of O. indicum on the migration of keratinocytes (HaCat cells) was determined by the scratch assay method. Scratches were made in confluent HaCaT monolayers. The scratches were treated with various concentrations (12.5–50 μg/ml) of extracts. The progress of the closure of the scratches was recorded after 24 h and the movement of the cells were observed. The *O. indicum* extract stimulated the migration of keratinocytes into the denuded area and resulted in faster closure of wound area compared with controls groups (Figure 3). The migration rates of HaCat cells that were treated with 50 μg/ml and 25 μg/ml concentrations were significantly higher (*p* < 0.05) compared to the negative control group. In addition, the migration rate of the HaCat cells that were treated with 50 μg/ml was significantly higher than the positive control. The effect of a range of concentrations of ethanolic leaves extracts on migration rate of HaCat cells are shown in Figure 3.

**FIGURE 4:**
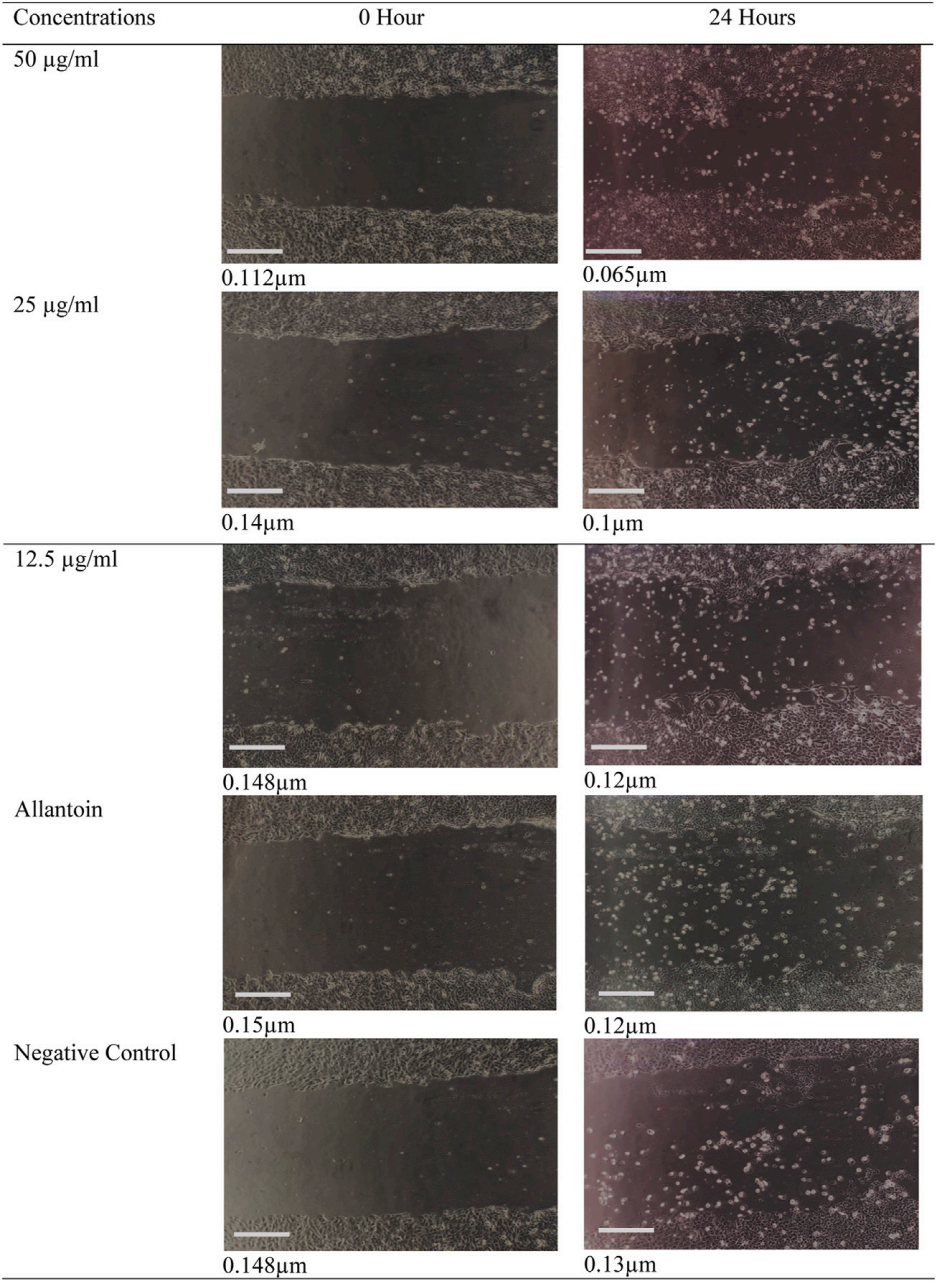
Digital image showing the effect of *Oroxylum indicum* leaves extracts on human keratinocyte cell migration in a scratch assay. Migrations of keratinocytes were photographed using light microscope fitted with digital camera with ×10 magnification. Bar = 1 cm.

The migration rates of the cells that were treated with 25 μg/ml and 12.5 μg/ml were not significantly (*p* < 0.05) different from positive control group. The present study indicates that the migration rate for the cell that was treated with the highest concentration of ethanolic extract (50 μg/ml) has highest migration rate compare to other concentrations. The migration of keratinocytes was seen to be more significant in the *O. indicum* extract-treated cells compared to vehicle-treated and allantoin-treated cells (Figure 4).

### The effect of ethanolic extract of *Oroxylum indicum* on *in vivo* excision wound model

**FIGURE 5:**
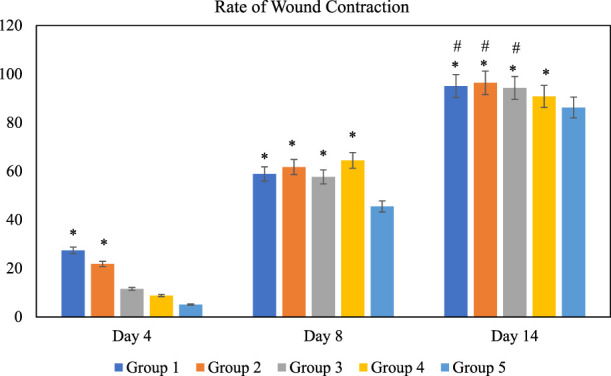
Rate of wound contraction for each group at different time intervals, days 4, 8, and 14 of the study. Notes: The symbol * denotes the values are significantly (*p* < 0.05) different between treated and negative control group and symbol # denotes the values are significantly (*p* < 0.05) different between treated and positive control group. Results are mean ± SD (n = 4) at (*p* < 0.05). Group 1: *O. indicum* (1%), Group 2: *O. indicum* (3%), Group 3; *O. indicum* (5%), Group 4: Positive control and Group 5: Negative control.

**FIGURE 6:**
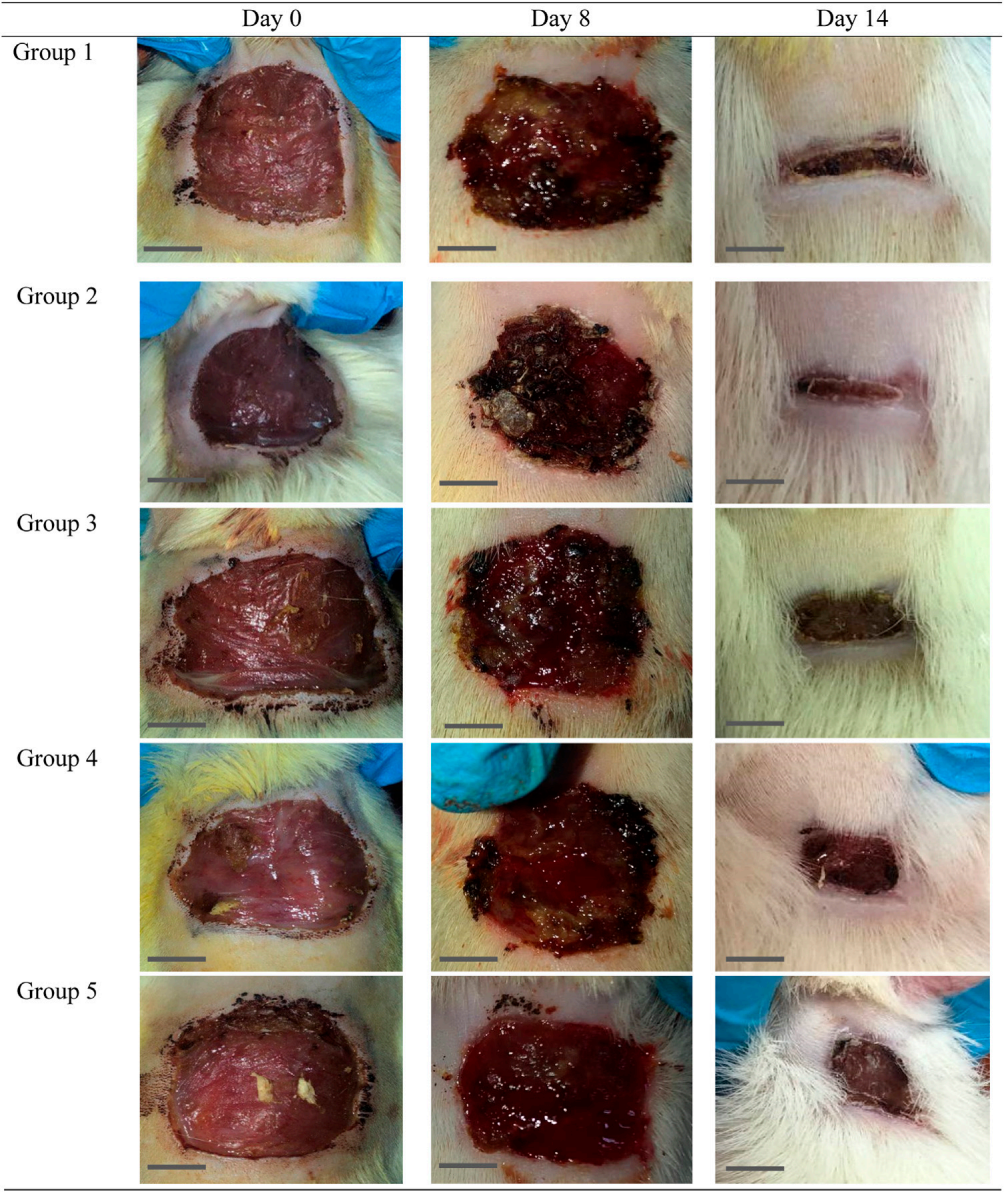
Gross morphology of the wound in the control and experimental groups. The wounds appeared dried and the scab was not uniformly on each wound started at day 4 of the study onwards. Bar = 1 cm.

The effect of *O. indicum* leaves extracts on excision wound model in albino rats is shown in Figure 5. A significant healing pattern with almost complete wound closure was observed in rats treated with different concentrations of the ethanol extract of *O. indicum,* whereas untreated animal groups lagged far behind. The increased rate of wound contraction lead to faster healing as confirmed by the increased healed area in the ethanolic extract-treated groups compared to the control ones (Figure 6).

## Discussion

*Oroxylum indicum* has long been used as a medicinal and culinary herb in many Asian countries, including Malaysia. The aerial parts or roots of this plant are used in traditional medicine in Malaysia for treatment of wounds, diarrhea, digestive problems, and control high blood pressure (Reduan et al., 2021). Although *O. indicum* is widely used in traditional medicines, there has been limited scientific investigation into the biological effects in relation to wound healing activity. Therefore, we evaluated the phytochemical constituents and wound healing properties of aqueous and ethanol extracts of *O. indicum* using *in vitro* and *in vivo* models.

In the present study *O. indicum* was evaluated for determination of total phenolic and flavonoids contents. The quantification revealed the ethanolic leaves extract of *O. indicum* showed higher total phenolic (164.9 mg GAE/g) and flavonoids contents (101.2 mg CE/g). The extract with aqueous showed lower quantity of phenols (30.1 mg GAE/g) and flavonoids (76.2 mg CE/g) that showed the ethanol as suitable for preparation of plant extracts with high yield of phenols and flavonoids from *O. indicum* leaves. Based on rich quantity of phenols and flavonoids observed in the preliminary investigation, *O. indicum* ethanol extract was subjected to chemical profiling using GC-MS and LC–MS/MS techniques. These analytical techniques have very high sensitivity and resolution, and are suitable for detailed characterization of plant extracts (Heinrich et al., 2020; Heinrich et al., 2022).

The phytochemical analysis of *O. indicum* leaf extract by GC-MS showed the identification of total 19 compounds. Of them, seven compounds were identified as esters. The Pentanoic acid, 2,2,4-trimethyl-3-carboxyisopropyl, isobutyl ester, Benzoic acid, 3,4-bis[(trimethylsilyl)oxy]-,trimethylsilyl ester, Hexadecanoic acid, trimethylsilyl ester, 9,12-Octadecadienoic acid (Z,Z)-, trimethylsilyl ester, à-Linolenic acid, trimethylsilyl ester, Octadecanoic acid, trimethylsilyl ester and hexadecanoic acid, 14-methyl were detected (Table 1). Pentanoic acid, 2,2,4-trimethyl-3-carboxyisopropyl, isobutyl ester and hexadecanoic acid shows anti-tumor, analgesic, antimicrobial and antioxidants activities (Kumar et al., 2019; Beschi et al., 2021; Madhavan et al., 2021). The following sugars, D-Fructose, 1,3,4,5,6-pentakis-O-(trimethylsilyl)-,O-methyloxime, d-Galactose, 2,3,4,5,6-pentakis-O-(trimethylsilyl)-,o-methyloxyme, (1E)-, Galacturonic acid, pentakis(trimethylsilyl)- and d-Galactose, 2,3,4,5,6-pentakis-O-(trimethylsilyl)-,o-methyloxyme, (1Z)- were detected. Three ether compounds such as Trimethylsilyl ether of glycerol, l-(-)-Arabitol, pentakis(trimethylsilyl) ether and Phytol, trimethylsilyl ether were also detected. Chrysin, bis(trimethylsilyl) ether, a flavone, was also detected in the GC–MS. Chrysin has been reported by others from different parts of *O. indicum* (Kumar et al., 2019). Chrysin, bis(trimethylsilyl) ether is a natural flavonoid that has been reported to have numerous biological activities such as hepatoprotective and antioxidant (Pushpavalli et al., 2010), cytoprotective and anti-inflammatory (Sulaiman et al., 2014; Deldar et al., 2018; Mani and Natesan, 2018), and cardiovascular protective effects (Farkhondeh et al., 2019).

The LC-TOF-MS/MS profile of *O. indicum* leaves extract revealed 12 major compounds. Among the identified compounds the number of flavonoids were in abundance. The second highest class of secondary metabolites were glycosides compounds followed by dicarboxylic acids. The current analysis showed the presence of number of phytochemicals which are reported with beneficial biological effects in previous studies. Compounds Orientin, Chrysin, Pinoquercetin, Cupressuflavone, Puerarin xyloside, Forsythiaside and Paederoside have been widely known to have biological activities such as antioxidant, antiviral, antibacterial, anti-inflammatory, and reduced oxidative stress and cardio protective effects (Qiufeng et al., 2004; Praveena et al., 2014; Zhao et al., 2018; Khalil et al., 2022). Chrysin, a natural flavonoid, has also a wide variety of biological activities such as anti-cancer (Ryu et al., 2017), wound-healing (Ansor, 2021; Kaparekar et al., 2021), anti-Inflammatory, and antioxidant activity (Beschi et al., 2021). Other compounds detected in the extract such as Pinoquercetin and Cupressuflavone have been reported to have an antioxidant property, anti-inflammatory, analgesic, and anti-bacteria activities (Quang et al., 2008; Al-Sayed et al., 2018). Together, the LC-TOF-MS/MS analysis of the *O. indicum* leaves extracts revealed important phytochemicals and this plant could be considered as good source of natural compounds.

Wound healing is a mechanism-based complex process of restoring cellular structures and tissue layers. The wound healing process involve different phases including contraction, the formation of epithelialization and fibrosis. The biological response regulating the body’s own cellular defense mechanisms contributes to the wound and its repair (Morales-González et al., 2022). In the present study, the wound healing effects of ethanolic extract of *O. indicum* were assessed in both *in vitro* and *in vivo* models.

The effects of the *O. indicum* extracts on wound closure were evaluated by *in vitro* wound scratch assay through observation of the migration rate of the HaCat cells (i.e. diameter of the wound area). The result of this study shows that the migration rate of cells that were treated with *O. indicum* extracts showed better migration rate compared with control. Furthermore, the wound closer time of extract-treated cells were faster when compared with other groups. Therefore, the *in vitro* wound-healing potential of *O. indicum* can be attributed to the contributions of bioactive compounds in the plant extract such as flavonoids, glycosides, alkaloids, and saponins (Praveena et al., 2014; Tran et al., 2015). These phytochemicals are usually responsible for the biological activity of medicinal plants.

The *O. indicum* extracts were further evaluated for its wound healing activity using excisional wound model (*in vivo*). The results of this study revealed that topical application of the ethanolic extract of *O. indicum* significantly (*p* < 0.05) increased wound healing effects by increasing the rate of wound contraction compared with control groups. Among the extract-treated groups, the highest contraction was observed in Group 2 (3% of the extract), whereas untreated animal showed lower contraction rate. The *O. indicum* extracts facilitated wound contraction significantly at all dose levels from 8^th^ day to 14^th^ day as compared to negative control. The phytochemical compounds present in the plant extract may contribute the wound-healing effect by eliminating infection and thus allowing initiation of natural tissue repair processes. These findings are in line with the study conducted by Samarath and Panda (2018) which reported that ethanolic extract has the highest rate of wound contraction in the excision wound and the existence of phytoconstituents such as flavonoids, saponins, phenols, and tannins, either alone or in combination, can have a synergistic impact on the wound healing process. Besides that, wound contraction also works as an indicator of active re-epithelialization, granulation, angiogenesis, fibroblast proliferation, keratinocyte differentiation, and proliferation activities occurred in the wound bed (Boakye et al., 2018). In addition, wounds of the rats in extract-treated groups showed more uniform scabs formation compared to positive and negative controls groups. The scab formation in extract-treated groups formed as early as day two of treatment compared to positive and negative groups. This indicated the presence of active blood clot formation and hemostasis occurred and faster in extract-treated groups.

Throughout the study the extract-treated groups showed no sign of pus formation which indicate there were no bacterial contamination or infection. These findings suggested that the extract may have antimicrobial activity, which fastens the inflammatory phase, and prepared the wound to enter the healing phase. The enhanced wound healing potency of various herbal extracts could be due to the antimicrobial and free radical-scavenging properties of the phytoconstituents present in the extract, and the faster wound healing process could be a individual or synergistic effects of bioactive molecules (Thakur et al., 2011). In our study, higher wound healing properties as exhibited by *O. indicum* could be due to its high content of phenolics and flavonoids in both the studied models (*in vitro* and *in vivo*).

## Conclusion

The present study demonstrated that *O. indicum* leaves extracts contains important phytochemicals. This study also demonstrated that animals treated with ethanol extract of *O. indicum* exhibited significant wound healing effects (*in vitro* and *in vivo*) and its healing potential may probably be as a result of the presence of a mixture of phytochemicals. Thus from this study it can be suggested that the ethanolic extract of *O. indicum* has wound healing potential and thereby it could be used as a natural wound-healing agent. Future research warrants identification of the individual compounds present in the *O. indicum* leaves responsible for its wound healing property and subsequent understanding of their mechanisms of bioactivities.

## Data Availability

The original contributions presented in the study are included in the article/supplementary material, further inquiries can be directed to the corresponding authors.

## Abbreviations

CE, Catechin equivalents; HaCaT, Human keratinocyte cells; IACUC, Institutional Animal Care and Use Committee; GAE, Gallic acid equivalents; GC-MS, Gas chromatography–mass spectrometry; LC-MS/MS, Liquid chromatography–mass spectrometry; NIST, National Institute of Standards and Technology; SD, Standard deviation; TFC, Total flavonoids content; TPC, Total phenolic content; UHPLC, Ultra-high-performance liquid chromatography.
